# Delivery of Pediatric Student-Led Speech and Language Therapy Services at a University Rehabilitation Clinic in Cyprus: Children Accessing Services

**DOI:** 10.3390/children12060671

**Published:** 2025-05-23

**Authors:** Theodora Papastefanou, Paris Binos, Despo Minaidou, Kakia Petinou, Costas A. Christophi, Eleni Theodorou

**Affiliations:** 1 Department of Rehabilitation Sciences, Cyprus University of Technology, Vragadinos 15, First Floor, Limassol 3036, Cyprus; paris.binos@cut.ac.cy (P.B.); costas.christophi@cut.ac.cy (C.A.C.);; 2 CIRCLE Laboratory, Department of Rehabilitation Sciences, Cyprus University of Technology, Limassol 3036, Cyprus

**Keywords:** speech and language therapy, university-led clinic, service accessibility, developmental speech and language disorders, Cyprus

## Abstract

**Background/Objectives:** Early identification and intervention in speech and language therapy (SLT) are essential for children’s academic, social, and emotional development. In Cyprus, barriers such as long waiting lists, financial constraints, and limited public awareness restrict access to SLT services. University-led clinics offer a promising alternative by providing affordable, accessible care while training future clinicians. This study aimed to examine the demographic profiles, referral pathways, and diagnostic patterns of children accessing services at a university-led SLT clinic. By documenting referral trends and diagnostic outcomes, this study offers preliminary insights into patterns of service use and potential access disparities in the Cypriot context. **Methods:** A retrospective analysis was conducted using records from 235 children, aged 0;7 to 15 years, assessed at the University Rehabilitation Clinic between 2015 and 2024. Data included age, gender, socioeconomic status (SES), bilingualism, referral source, and diagnostic outcomes. Diagnoses were classified using Bishop et al.’s (2016) framework. **Results:** Significant associations were identified between age, parental education, referral source, and diagnostic category. Older children (9;1–12 years) demonstrated a markedly increased likelihood of receiving a developmental language disorder (DLD) diagnosis. Higher parental education levels and referrals from teachers or parents were also predictive of DLD and other communication impairments. Bilingualism was not a significant predictor of diagnostic category. **Conclusions:** The findings suggest that university-led clinics may serve as an important access point for underserved populations in Cyprus. This study provides preliminary evidence concerning demographic and referral factors that can inform outreach strategies and future service planning.

## 1. Introduction

Effective communication and language skills are essential for children’s academic, social, and emotional development. Early identification and intervention for speech and language difficulties play a crucial role in preventing future learning challenges and improving overall well-being [1,2]. For instance, Conti-Ramsden et al. (2015) [3] found that children with language impairments are at greater risk for social and emotional difficulties during adolescence, impacting their long-term well-being. Similarly, Snowling et al. [2] highlighted that early language problems are predictive of later reading difficulties and can have cascading effects on academic achievement and mental health.

Despite the critical importance of early intervention, access to speech and language therapy (SLT) services varies across different contexts, with significant disparities (e.g., socioeconomic, geographic) in service provision [4,5]. Although there is limited peer-reviewed research specifically examining SLT service access in Cyprus, international evidence consistently highlights barriers such as long public-sector waiting lists, financial constraints in accessing private therapy, and geographical limitations affecting rural populations [6,7]. University-led SLT clinics have been proposed as a potential solution to address these service gaps, offering accessible and affordable interventions [6,8]. Internationally, university-affiliated clinics have demonstrated success in improving access to SLT services. For example, in Australia, university clinics have provided low-cost therapy options, while also serving as valuable training environments for students, thereby expanding service capacity and reducing waiting times [6]. Similarly, in the United States, university clinics have played a pivotal role in assisting underserved populations, including rural and low-income communities, by providing sliding scale fees and outreach services [9]. These examples have illustrated the potential for university-led clinics to complement public and private services, improving access and equity in SLT provision.

University-led clinics can enhance service provision while providing valuable training opportunities for future clinicians [9]. Research has suggested that such clinics contribute to increasing service availability and providing alternative intervention pathways, particularly where public and private services are insufficient [6,9]. However, little is known about how university-led SLT clinics function in the Cypriot context, particularly in terms of accessibility and service reach. This study addresses this gap by focusing on one such clinic to examine referral pathways, demographics, and service accessibility.

### 1.1. Demographics and Referral Pathways

Understanding the demographics of children accessing SLT services is crucial for assessing equity, planning interventions, and identifying systemic gaps. In Cyprus, the population served by SLT services is heterogeneous, exhibiting variations in age, gender, socioeconomic status (SES), and linguistic background. However, comprehensive demographic data remain limited, restricting the development of targeted intervention programs.

Socioeconomic and educational background have been identified as key driving factors impacting SLT access and outcomes. Children from higher-income families are more likely to access private SLT services, whereas those from lower-income backgrounds often have limited options and face delays due to the waiting lists encountered in the national health services [4]. Similarly, parental education level plays a significant role in shaping perceptions of speech and language issues and influencing help-seeking behavior. Moreover, bilingual children face unique challenges, as they are often misdiagnosed or underdiagnosed due to misconceptions about typical bilingual development and limitations in assessment tools [7,10].

Gender differences in referral patterns have also been documented. Boys are more frequently referred for SLT services compared to girls, potentially due to increased awareness of expressive language difficulties in male children [11]. Additionally, referral sources play a critical role in determining access to services. In Cyprus, most referrals originate from parents, educators, and pediatricians [4]. However, awareness of university-led clinics as viable service providers remains low, limiting their utilization.

Recent studies underscore the importance of communication between SLTs and parents/guardians (P/Gs) in improving service quality. Voniati et al. [12] examined the communication practices between SLTs and P/Gs of children with developmental language disorders (DLDs) in Cyprus. While in-person communication was the most common communication method, only 48.7% of P/Gs reported feeling well-informed about their child’s progress and goals. This suggests a pressing need for enhanced parental engagement and transparency in SLT services.

Factors such as the age of referral, gender ratios, and specific language disorders significantly shape therapy needs [13,14]. For example, Broomfield and Dodd [13] documented the prevalence of articulation and phonological disorders in caseloads, while Harel et al. (1996) [15] noted a higher proportion of boys receiving therapy and a predominance of expressive language difficulties. Keegstra et al. (2007) [14] explored challenges in sentence production and comprehension, underscoring the diverse linguistic profiles of children in therapy. These findings have highlighted the necessity of tailored assessment and intervention strategies, particularly in countries like Cyprus, where data gaps limit the ability to effectively address children’s diverse communication needs.

### 1.2. Types of Speech and Language Disorders and Service Accessibility

According to Bishop et al.’s [16] causal framework, speech and language disorders in children are conceptualized across overlapping categories, acknowledging both primary and secondary language difficulties. The framework delineates speech sound disorders (SSDs), developmental language disorder (DLD), language disorders associated with biomedical conditions, and other communication impairments such as fluency and pragmatic language disorders. This model is particularly valuable in clinical and research contexts, as it highlights the multifactorial nature of communication disorders and the frequent co-occurrence of speech and language difficulties [16]. Such comprehensive classification systems are essential for guiding targeted interventions and service planning, particularly in settings where early identification and differential diagnosis are critical for improving services [17]. Recent research continues to emphasize the importance of frameworks like Bishop’s for informing best practice in regards to assessment and intervention, supporting clinicians in making accurate, individualized decisions in increasingly diverse and complex caseloads [18].

In Cyprus, a study by Theodorou et al. [19] identified mixed receptive–expressive language disorders as the most common diagnosis among children with DLD. Diagnosis typically occurred at an average age of seven years, often occurring later than is optimal for early intervention. Interventions were primarily individualized and conducted biweekly, with no standardized duration, underscoring the need for defining uniform service guidelines.

Access to timely intervention remains a critical concern. Long waiting lists in the public sector and high costs in the private sector create significant disparities [4]. University-led SLT clinics have emerged as a promising alternative by providing affordable care, while also serving as training centers for student clinicians. Previous research has indicated that children from lower-income backgrounds are more likely to experience delays in receiving therapy due to financial constraints [9]. University-led clinics provide an affordable alternative, yet they face challenges such as inconsistent scheduling due to student availability and limited awareness among families. Studies from other countries identified similar findings, showing that university-based clinics can help bridge service gaps, whereas sustainability remains a concern due to reliance on student clinicians and academic cycles.

### 1.3. Assessment Methods and Linguistic Considerations

The linguistic landscape in Cyprus is shaped by diglossia, as children grow up exposed to both Standard Modern Greek and the regional dialect, Cypriot Greek. These varieties differ significantly in phonology, syntax, and morphology [20,21,22]. This environment, described as bilectalism [23], complicates SLT assessments, particularly when tools standardized on monolingual populations are applied to bilectal children.

Misclassification of typical dialectal features as disordered speech patterns is a common issue. For instance, clitic placement in Cypriot Greek can diverge from Standard Greek norms, leading to false-positive diagnoses of morphosyntactic impairment (Agouraki, 1997) [24]. To address this issue, researchers advocate for the development of dialect-sensitive assessment tools, tailored to the linguistic realities of Cypriot children [25,26].

The lack of a centralized, standardized protocol for speech and language assessment in Cypriot Greek further complicates diagnoses. Theodorou et al. [19] stressed the urgent need for culturally and linguistically appropriate diagnostic measures to reduce variability and enhance service consistency. University-led clinics can play a central role in piloting and validating such tools within real-world clinical contexts.

### 1.4. Present Study

This study provides a detailed description of the caseload characteristics of all children referred to the University Rehabilitation Clinic at the Cyprus University of Technology for the assessment of speech and language difficulties between September 2015 and April 2024. The study examined population factors (namely referral source, age, gender, SES, and bilingualism) and explored the relationship between these factors and speech and language diagnoses that the children received. The research questions were as follows:

1. What demographic factors (namely age, gender, SES, bilingualism) and referral-related factors were evident in children with speech and language difficulties?
2. What demographic and referral-related factors are associated with the likelihood of different speech and language disorder diagnoses in children referred to a university-led speech and language therapy clinic in Cyprus?

## 2. Methods

### 2.1. Participants

The study cohort comprised 235 children, ranging in age from 7 months (0;7 years) to 15 years, who attended the University Rehabilitation Clinic between September 2015 and April 2024. Parental consent was obtained for all participants. The referral age for each child was determined by calculating the time from their date of birth to the date of their first assessment. Participants were divided in five age groups: Group 1 consisted of the ages 0;7–3, Group 2, the ages 3;1–6, Group 3, the ages 6;1–9, Group 4, children 9;1–12 years old, and Group 5, the ages 12;1–15 years. The distinction between age groups in the study was made to reflect the critical stages of language and speech development, which are well-documented in the literature. Each stage aligns with significant linguistic, cognitive, and social milestones that influence the assessment and intervention needs for children with speech and language disorders.

### 2.2. University Rehabilitation Clinic

The University Rehabilitation Clinic of the Cyprus University of Technology is the first University Clinic in Cyprus to serve as a clinical, teaching and research facility for allied health professionals. Currently, the aim is to provide high-quality rehabilitation services, while also contributing to the academic and professional development of students in the field of speech and language therapy. It is important to note that therapy sessions at the University Rehabilitation Clinic are conducted by third-year SLT students under the direct supervision of certified clinicians. Sessions typically follow individualized intervention plans based on initial assessments and include evidence-based practices targeting speech, language, and communication goals. The University Rehabilitation Clinic adheres to the rules and regulations outlined in the Code of Ethics of the Cypriot Speech and Language Pathologists Association. Consistent with practices in other speech therapy centers, the clinic is dedicated to providing appropriate care characterized by quality, control, and prevention. Essential data, constituting a minimum set, are systematically collected for all cases involving the provision of speech therapy. The clinic’s operational procedures include a dedicated file which is established for each case, encompassing pertinent information such as medical and language history, assessment plans, scoring sheets, and requisite consent forms. Throughout the duration of service provision at the clinic, meticulous record-keeping is ensured, with all forms being cataloged within individual files and securely stored in cabinets with restricted access. This protocol effectively addresses the full spectrum of ethical considerations, as outlined by the Cyprus Association of Registered Speech–Language Pathologists.

Importantly, the clinic operates in accordance with the academic calendar of the university. As such, referral intake and assessment availability may fluctuate during holiday periods or academic breaks, potentially introducing seasonal variability into the case distribution. While not the primary focus of this study, this operational structure may influence the flow and consistency of referrals throughout the year.

### 2.3. Data Collection and Recording Process

Children were assessed individually by speech and language therapy students as part of their clinical training. All assessments were conducted by third-year SLT students under the direct supervision of a certified and experienced speech and language therapist. While supervisors were present during assessments to ensure procedural fidelity, students used a standard set of tools and followed clinic guidelines. However, due to the training context and evolving clinic practices, some variation in assessment procedures may have occurred. Each child underwent a comprehensive speech and language assessment that included both standardized and non-standardized measures tailored to their linguistic and developmental profiles. Among the standardized tools administered were the Peabody Picture Vocabulary Test–Revised (Greek version) [27], as well as the Greek adaptations of the Renfrew Word Finding Test [28] and the Renfrew Bus Story Test [26,29]. Theodorou et al. [26] have demonstrated that these assessments, among others, possess diagnostic validity for use with Greek-Cypriot children. Inter-rater agreement between two researchers regarding transcription was 80%. Diagnostic and service-related decisions showed consistent agreement between raters, suggesting generally high reliability across coding categories. Using Bishop et al.’s [16] classification, these disorders were broadly categorized into speech sound disorders (SSDs), developmental language disorder (DLD), language disorders associated with biomedical conditions (LDs), and other speech, language, and communication difficulties, ensuring a structured approach to identifying and understanding the diverse challenges faced by the children. Children who presented with overlapping features of SSDs and DLD or other comorbidities were not excluded from the analysis. Instead, they were classified under composite category (Other) [16,30]. According to the CATALISE framework [31], comorbidities refer to co-occurring difficulties in cognitive, sensorimotor, or behavioural domains that may accompany developmental language disorder (DLD). These include conditions such as ADHD, developmental coordination disorder (DCD), dyslexia, speech impairments, emotional and behavioral disorders, and limitations in adaptive functioning. While such comorbidities can influence the presentation and intervention outcomes of DLD, their causal relationship to the language disorder remains unclear.

Two research assistants meticulously transferred the requisite data of the children and built an electronic database to facilitate subsequent statistical analysis. Demographic and environmental characteristics were collected, including age, gender, referral source, children’s diagnoses, socioeconomic background (SES), and bilingualism status.

Socioeconomic status (SES) was defined according to the parents’ level of education, which was classified into five groups: 1 = elementary school, 2 = secondary school, 3 = high school, 4 = undergraduate degree, and 5 = postgraduate degree. The first two levels were grouped as low-SES, the third as middle-SES, and the last two as high-SES. While we acknowledge that this approach provides only a partial representation of SES, it was the only consistently available indicator across all clinic records. Income, employment status, and geographic or neighborhood indicators were not systematically collected as part of the assessment protocol during the study period. However, parental education serves as a key indicator of SES and is a strong, independent predictor of children’s academic performance [32,33,34].

With regard to children growing up in a multilingual environment, we coded this variable into two levels. The first level included the dialectal Cypriot Greek speaking children, and the second one included children who were exposed to two linguistic varieties, specifically, their maternal/heritage language (e.g., Russian, English, Romanian) and the dominant societal linguistic varieties, i.e., the Cypriot Greek variety and Standard Modern Greek.

### 2.4. Design

This study employed a retrospective study design, analyzing data collected during the provision of speech and language therapy (SLT) services at the University Rehabilitation Clinic of the Cyprus University of Technology. The clinic serves as both a training facility for speech and language therapy students and a community resource for children with suspected or diagnosed speech and language disorders. A database was created to compile existing assessment and diagnostic data from children who attended the clinic, allowing for an in-depth examination of demographic variables, referral sources, and patterns in speech and language diagnoses.

### 2.5. Statistical Analysis

We conducted a descriptive and inferential statistical analysis using IBM SPSS Statistics (Version 27.0) to examine the distribution and associations among key variables. Frequency distributions were calculated for categorical variables, including gender, age of referral, source of referral, socioeconomic status (SES), bilingualism status, and speech and language diagnosis. To explore potential associations between these variables, Chi-square (*χ*^2^) tests of independence were performed to assess statistical significance. These analyses aimed to identify trends in diagnostic patterns across different demographic groups and referral sources, providing a comprehensive understanding of caseload characteristics in the studied population.

## 3. Results

To explore potential associations, Chi-square (*χ*^2^) tests were used for bivariate analyses (see the Table 1 and Table 2), followed by a multinomial logistic regression to examine how demographic and referral factors predicted diagnostic outcomes. The reference category was speech sound disorders (SSDs), and comparisons were made against three other categories: developmental language disorder (DLD), language disorders associated with biomedical conditions (LD), and other diagnoses (see the Table 3).

Categorical predictors (e.g., age group, gender, SES level, referral source) were dummy-coded prior to model entry. The model was assessed using Nagelkerke’s pseudo R^2^, and likelihood ratio tests were used to evaluate overall model fit. Multicollinearity was assessed using the variance inflation factor (VIF), and no variables exceeded the conventional threshold of 2.5. Model diagnostics and confidence intervals were calculated to enhance the interpretability and reliability of the findings.

### 3.1. Age

The majority (52.8%) were aged 3;1–6 years, followed by 6;1–9 years (26%). Notably, SSD was most prevalent in the 3;1–6 age group (45.4%) (Table 1), while DLD became more prominent in older children, peaking at 75% in the 9;1–12 age group (*χ*^2^(12) = 45.643, *p* = 0.003). These findings suggest that SSD is primarily identified in preschool years, whereas DLD emerges more clearly as children advance in school (Table 2).

### 3.2. Gender

Table 1 shows that boys accounted for 63.4% of the sample, consistent with previous findings showing higher referral rates for males. However, no significant gender differences were found in diagnostic distribution (*χ*^2^(3) = 28.48, *p* = 0.05). SSD was slightly more common in girls (37.2%) than boys (29.5%), whereas LD was more frequently diagnosed in boys (22.2%) compared to girls (16.3%) (Table 2).

### 3.3. Socioeconomic Status (SES)

Parental education, used as a proxy for SES, was significantly associated with diagnostic categories (*χ*^2^(12) = 34.59, *p* < 0.001). Table 2 shows that children from lower SES backgrounds (primary/secondary school) had a higher prevalence of SSD (45.5–100%), whereas DLD was more frequently diagnosed in middle SES groups (high school: 53%). The university-educated group had a more balanced distribution, while the postgraduate group showed higher rates of SSD (37.9%) and DLD (31%).

### 3.4. Monolingual vs. Bilingual Status

Table 1 shows that most children (83.4%) were monolingual, with bilingual children comprising 16.6% of the sample. No significant association was found between bilingualism and language disorder type (*χ*^2^(3) = 13.45, *p* = 0.04) (Table 2). Diagnostic patterns were similar across both groups, with SSD being the most frequent diagnosis among both monolinguals (33.2%) and bilinguals (28.2%).

### 3.5. Source of Referrals

Table 2 presents that the referral patterns varied significantly across professionals (*χ*^2^(12) = 28.384, *p* = 0.04). Doctors referred more cases of SSD (39%), teachers primarily referred children with DLD (43.4%), and speech–language therapists identified a mix of SSD (34.3%) and LD (34.3%). Parents demonstrated broad awareness of speech and language difficulties, referring for SSD (33.9%), DLD (25.4%), and LD (28.8%) at similar rates. Other professionals (e.g., psychologists, occupational therapists) provided diverse referrals across diagnostic categories.

### 3.6. Associations Between Demographic Variables and SLT Diagnoses

A multinomial logistic regression was conducted to examine the relationship between demographic and referral factors and the likelihood of receiving different diagnostic classifications. The model showed a Nagelkerke pseudo R^2^ of 0.384, suggesting a moderate level of explanatory power. The likelihood ratio Chi-square test was statistically significant (*χ*^2^(48) = 83.42, *p* < 0.001), indicating that the model performed better than a null model. No multicollinearity was detected among predictors (all VIFs < 2.0).

Table 3 presents the odds ratios (OR), 95% confidence intervals (CI), and *p*-values for each diagnostic category relative to the reference category (SSD).

### 3.7. DLD

Age was a significant predictor of DLD. Children aged 6;1–9 years were 2.42 times more likely to be diagnosed with DLD compared to those aged 0;7–3 years, *p* = 0.01. The likelihood increased further for children aged 9;1–12 years, who were 5.82 times more likely to receive a DLD diagnosis, *p* = 0.002. Children from high school-educated families also showed increased odds of receiving a DLD diagnosis (OR = 1.96), *p* = 0.03, compared to those from lower-educated backgrounds. Teacher referrals were strongly associated with DLD diagnoses (OR = 2.51), *p* = 0.004, while parent referrals were also significant predictors (OR = 1.73), *p* = 0.02.

### 3.8. LD

Girls were significantly less likely to be diagnosed with LD compared to boys (OR = 0.64), *p* = 0.03. Children from university-educated families were more likely to be diagnosed with LD (OR = 1.46), *p* = 0.05. No other predictors reached statistical significance in this diagnostic category.

### 3.9. Other Diagnoses

University-level parental education was significantly associated with an increased likelihood of receiving an “Other” diagnosis (OR = 1.76), *p* = 0.01. Both teacher (*p* = 0.02) and parent referrals (*p* = 0.04) were significantly associated with diagnoses in this category. Age (6;1–9 years) was also a significant predictor (OR = 1.40), *p* = 0.04.

### 3.10. Non-Significant Predictors

Bilingualism was not a statistically significant predictor in any diagnostic category, suggesting that bilingual children were no more or less likely to receive any specific diagnosis than were monolingual children.

## 4. Discussion

This study has provided the first comprehensive exploration of speech and language diagnoses among Cypriot Greek-speaking children referred to a university-led speech and language therapy (SLT) clinic in Cyprus. By analyzing caseload characteristics—including age, gender, socioeconomic status (SES), bilingualism, and referral source—we have gained insights into the demographic and contextual factors influencing diagnostic trends. Our findings have both aligned with and diverged from existing international studies [6,7], offering a localized perspective with broader implications for clinical practice and policy development.

### 4.1. Age and Diagnosis Trends

Our findings have underscored the developmental nature of speech and language disorders. Younger children (aged 0;7–3 years) have been more commonly diagnosed with LD, supporting existing research that has emphasized the importance of early language development in predicting later academic success [2,35]. SSDs have been more prevalent in preschool-aged children (3;1–6 years), reflecting typical patterns in the acquisition of speech sounds [7].

As children have aged, DLD has become increasingly evident. This trend has mirrored previous studies indicating that DLD often remains under-identified in early years and becomes more apparent when children encounter complex academic and linguistic tasks in school [2,11]. Our findings have reinforced the need for sustained monitoring of language development beyond preschool, particularly as DLD symptoms have persisted into later childhood and adolescence [16].

### 4.2. Gender Differences

In terms of gender, our data have shown that boys have been more likely to be referred and diagnosed with LD, consistent with research suggesting that boys have been overrepresented in SLT referrals [13]. However, within our sample, girls have also been diagnosed with SSD and DLD, suggesting that while boys may attract earlier attention, language difficulties in girls may have been under-identified or misunderstood. This discrepancy has highlighted the need for gender-sensitive diagnostic practices to ensure equity in referral and intervention.

### 4.3. Socioeconomic Influences on Diagnosis

Our analysis has revealed that children from lower SES backgrounds, particularly those whose parents have completed only a high school education, have been more likely to be diagnosed with DLD. This result supports the findings of Bishop et al. [16] and Hoff [36], who have emphasized the role of SES in shaping early language development and access to resources. In contrast, children from university-educated families have exhibited a broader distribution of diagnoses, including LD and other communication impairments, suggesting greater awareness and more proactive engagement with SLT services.

These findings have aligned with research by Dew et al. [9] and Morgan et al. [5] (2019), which identified access barriers—such as long public-sector waiting lists and financial constraints—as disproportionately affecting families from lower SES backgrounds. Our study has reinforced the importance of targeted outreach and early screening initiatives in these communities to ensure timely intervention.

### 4.4. Bilingualism and Diagnostic Patterns

Although bilingualism has not emerged as a statistically significant predictor of diagnosis, a higher proportion of bilingual children have been diagnosed with DLD than with other disorders. This result aligns with research by Peña et al. [37], which has noted that bilingual children may present more visible language challenges, particularly when assessed using monolingual norms. However, our findings have also supported the findings of Paradis et al. [38], who have argued that these differences often reflect typical bilingual development rather than underlying disorders.

The linguistic context of Cyprus—characterized by bilectalism involving Cypriot Greek and Standard Modern Greek—has further complicated assessment [23]. Without dialect-sensitive assessment tools, there has been a risk of misdiagnosing linguistic variation as a disorder. Thus, our study has emphasized the need for culturally and linguistically appropriate evaluation protocols tailored to the Cypriot context.

### 4.5. Referral Pathways and Identification

Consistent with previous studies [4,13], parents and teachers have been the primary sources of referrals. Notably, teachers have been more likely to identify children with DLD, likely due to the increased academic language demands in school settings. Parents, on the other hand, have been more likely to initiate referrals for SSD. This division of referral types has reflected differences in the environments where various speech, language and communication needs are most noticeable—home vs. classroom.

These findings have illustrated the importance of equipping both teachers and parents with the knowledge and tools to recognize early signs of communication difficulties. Furthermore, raising awareness about the availability and role of university-run clinics could expand referral pathways and increase access to early diagnosis.

### 4.6. Clinical and Policy Implications

This study offers important implications for clinical practice and service delivery. First, age-related trends have pointed to the necessity of longitudinal monitoring of children’s language development, particularly into the school years, when more complex language difficulties become apparent. Second, SES-related disparities in diagnosis have called for systemic interventions, including universal early screening and subsidized services for low-income families.

University-led SLT clinics, such as the one examined in this study, have been uniquely positioned to address these needs. As suggested by Dew et al. [9], these clinics have offered affordable services and served as training grounds for future clinicians. However, their effectiveness has depended on consistent scheduling, robust supervision, and broader public awareness. Integrating these clinics more fully into the national healthcare system could expand access and improve service equity.

The role of teachers in identifying DLD has also warranted attention. Professional development programs focused on speech and language development could strengthen early detection and improve referral accuracy. Similarly, improving communication between SLTs and families—as highlighted in recent findings by Voniati et al. [12]—has been essential to ensure that parents are well-informed and actively engaged in their child’s therapy.

Furthermore, the findings of this study have significant implications for policy development. Enhancing the availability and quality of diagnostic and intervention services for children with speech, language, and communication needs is critical to advancing national objectives regarding both education and public health. Importantly, we advocate for the adoption of standardized, evidence-based frameworks—such as the CATALISE framework [16]—to guide the systematic evaluation of SLT service provision across a range of settings, including public healthcare institutions and private practices. The implementation of such frameworks would facilitate ongoing monitoring of service effectiveness, promote consistency in care delivery, and ensure that assessment results are comparable across different settings. This, in turn, would enhance communication among professionals, both within and between service contexts.

### 4.7. Limitations and Future Research

While this study provides valuable insights into diagnostic trends and service access in a university-led speech and language therapy clinic, several limitations must be acknowledged. First, the retrospective nature of the dataset constrained the availability of detailed clinical information, including disorder severity, intervention outcomes, and contextual family factors such as parental language practices. Second, the study was based on data from a single university-based clinic, which may limit the generalizability of findings to broader clinical or geographic settings. The clinic’s operation, closely tied to the academic calendar, may have also introduced seasonal variability in referral and assessment patterns. Future research should collect data from a wider range of diverse clinical settings—including urban and non-urban areas, as well as private clinics and hospitals—to enhance the generalizability of the findings [39]. Moreover, incorporating detailed clinical variables—such as disorder severity, treatment outcomes, and family-contextual factors like parental language practices—would provide a more comprehensive understanding of diagnostic trends and service delivery [40]. Additionally, longitudinal designs that track clients over time could help clarify how clinic operations (e.g., alignment with academic calendars) affect referral patterns and continuity of care [41].

Another limitation is the absence of therapy outcome data. While the scope of the study is to highlight important trends in access and diagnosis, and it does not allow for the evaluation of intervention effectiveness or child-level progress, future research should adopt prospective designs to track therapy goals, progress, and outcomes over time.

Regarding SES, we used parental education as a proxy due to data availability. However, we acknowledge that the educational background of the parents does not fully capture economic stability, especially in contexts of underemployment or wage variability. A more composite SES index—including household income, employment, and neighborhood-level indicators—would offer a more accurate view of structural influences on service access. Future work should also consider practical access barriers such as transportation and the affordability of private services.

Finally, future research should prioritize prospective longitudinal designs to evaluate the long-term outcomes of children receiving services through university-led clinics, particularly among those from diverse SES and linguistic backgrounds. Culturally responsive and dialect-sensitive assessment tools, especially for bilectal and bilingual populations, are essential for improving diagnostic accuracy and equity in service provision.

## 5. Conclusions

This study represents the first systematic investigation into the diagnostic patterns of speech and language disorders among Cypriot Greek-speaking children referred to a university-led SLT clinic in Cyprus. Through a comprehensive analysis of demographic and contextual variables—such as age, gender, SES, bilingualism, and referral source—we identified key diagnostic trends that align with findings from international research [6,7], while also offering evidence regarding the local clinical landscape. In addition, the study contributes to the emerging literature on alternative SLT delivery models, particularly the role of university-led clinics in addressing service gaps and supporting underserved populations [8,9].

Our findings have highlighted the developmental trajectory of speech and language disorders, including the early identification of SSD during the preschool years and the increasing identification of DLD in later childhood. Socioeconomic disparities emerged as significant factors influencing access to services, with children from lower SES backgrounds more frequently diagnosed with DLD. Although bilingualism was not a statistically significant predictor of diagnostic category, the findings have underscored the importance of implementing culturally and linguistically responsive assessment protocols tailored to the Cypriot context [26]. The prominent role of referral sources—particularly parents and teachers—has further emphasized the importance of public awareness and interdisciplinary collaboration in facilitating the timely and accurate identification of children with speech, language, and communication needs. Importantly, despite its limited scope, the university clinic setting demonstrated its potential as a cost-effective and accessible model for both service delivery and clinician training.

Future research should expand this research by incorporating a broader range of clinical settings and employing longitudinal designs that can better capture the evolving nature of co-occurring conditions such as SSD and DLD. Future policies should also prioritize equitable access to early screening and intervention, particularly for families from lower socioeconomic backgrounds. Addressing these challenges will require a coordinated and systemic response. Key strategies include implementing universal early screening programs, developing culturally and linguistically appropriate diagnostic tools, providing targeted training for clinicians, and expanding the reach of university-led clinics. Strengthening early identification and referral mechanisms—especially for at-risk and underserved populations—is critical to the development of a more inclusive, equitable, and responsive SLT service model in Cyprus.

Moreover, these efforts carry broader policy implications. Enhancing diagnostic and intervention services for children with speech, language, and communication needs can make a significant contribution to national strategies aimed at improving both educational and health outcomes. We advocate for the adoption of a structured approach to evaluating SLT service provision across diverse settings, including hospitals and private practices, guided by evidence-based frameworks such as the CATALISE framework [16]. Such frameworks can support ongoing service monitoring and help identify systemic gaps in diagnosis and intervention.

## Figures and Tables

**Table 1:** Overall shares of each group and condition.

Group		N = 235	Percentage (Overall)	SSD	DLD	LD	Other
Age	0;7–3	30	12.8%	1.7%	1.7%	6.4%	3.0%
	3;1–6	124	52.8%	23.4%	9.4%	11.5%	8.5%
	6;1–9	61	26.0%	5.5%	13.2%	2.1%	5.1%
	9;1–12	12	5.1%	0.4%	3.8%	0.0%	0.9%
	12;1–15	8	3.4%	1.3%	2.1%	0.0%	0.0%
Gender	Boys	149	63.4%	18.7%	18.3%	14.0%	12.3%
	Girls	86	36.6%	13.6%	11.9%	6.0%	5.1%
SES	Primary school	2	0.9%	0.9%	0.0%	0.0%	0.0%
	Secondary school	11	4.7%	2.1%	0.9%	0.9%	0.9%
	High school	49	20.9%	3.8%	11.1%	3.8%	2.1%
	University	144	61.3%	20.9%	14.5%	13.6%	12.3%
	Postgraduate diploma	29	12.3%	4.7%	3.8%	1.7%	2.1%
Bilingualism status	Monolinguals	196	83.4%	27.7%	24.7%	17.4%	13.6%
	Bilinguals	39	16.6%	4.7%	5.5%	2.6%	3.8%
Source of referral	Doctors	36	15.3%	6.0%	3.8%	2.6%	3.0%
	Parents	59	25.1%	8.5%	6.4%	7.2%	3.0%
	Teachers	53	22.6%	5.1%	9.8%	2.1%	5.5%
	Speech and Language Therapists	35	14.9%	5.1%	3.4%	5.1%	1.3%
	Other professionals	52	22.1%	7.7%	6.8%	3.0%	4.7%
Total			100.0%	32.3%	30.2%	20.0%	17.4%

**Table 2:** Summary of demographic and referral characteristics of children with speech and language disorders (breakdown of the conditions for each group and subgroup).

Group		N = 235	SSD		DLD		LD		Other *		*p*-Value
Age	0;7–3	30	4	13.3%	4	13.3%	15	50.0%	7	23.3%	0.001
	3;1–6	124	55	44.4%	22	17.7%	27	21.8%	20	16.1%	
	6;1–9	61	13	21.3%	31	50.8%	5	8.2%	12	19.7%	
	9;1–12	12	1	8.3%	9	75.0%	-	0.0%	2	16.7%	
	12;1–15	8	3	37.5%	5	62.5%	-	0.0%	-	0.0%	
Gender	Boys	149	44	29.5%	43	28.9%	33	22.1%	29	19.5%	ns.
	Girls	86	32	37.2%	28	32.6%	14	16.3%	12	14.0%	
SES	Primary school	2	2	100%	-	0.0%	-	0.0%	-	0.0%	0.04
	Secondary school	11	5	45.5%	2	18.2%	2	18.2%	2	18.2%	
	High school	49	9	18.4%	26	53.1%	9	18.4%	5	10.2%	
	University	144	49	34.0%	34	23.6%	32	22.2%	29	20.1%	
	Postgraduate diploma	29	11	37.9%	9	31.0%	4	13.8%	5	17.2%	
Bilingualism status	Monolinguals	196	65	33.2%	58	29.6%	41	20.9%	32	16.3%	ns.
	Bilinguals	39	11	28.2%	13	33.3%	6	15.4%	9	23.1%	
Source of referral	Doctors	36	14	38.9%	9	25.0%	6	16.7%	7	19.4%	0.04
	Parents	59	20	33.9%	15	25.4%	17	28.8%	7	11.9%	
	Teachers	53	12	22.6%	23	43.4%	5	9.4%	13	24.5%	
	Speech and Language Therapists	35	12	34.3%	8	22.9%	12	34.3%	3	8.6%	
	Other professionals	52	18	34.6%	16	30.8%	7	13.5%	11	21.2%	

* Other professionals include other professionals such as psychologists and occupational therapists.

**Table 3:** Multinomial logistic regression predicting speech and language diagnosis category (reference category: SSD).

Predictor	OR (DLD)	95% CI (DLD)	*p* (DLD)	OR (LD)	95% CI (LD)	*p* (LD)	OR (Other)	95% CI (Other)	*p* (Other)
Age (Ref. = 0;7–3 years)									
3;1–6 vs. Ref.	1.52	[0.99, 2.33]	0.05 *	1.23	[0.89, 1.69]	0.08	1.20	[0.85, 1.70]	0.10
6;1–9 vs. Ref.	2.42	[1.21, 4.83]	0.01 **	0.89	[0.50, 1.56]	0.30	1.40	[1.01, 1.93]	0.04 *
9;1–12 vs. Ref.	5.82	[1.90, 17.84]	0.002 **	0.52	[0.18, 1.46]	0.25	1.34	[0.88, 2.04]	0.07
Gender (Ref. = Boys)									
Girls vs. Ref.	0.75	[0.51, 1.09]	0.12	0.64	[0.42, 0.98]	0.03 *	0.83	[0.56, 1.23]	0.22
SES (Ref. = Primary/Secondary)									
High School vs. Ref.	1.96	[1.07, 3.60]	0.03 *	0.74	[0.38, 1.43]	0.18	0.80	[0.44, 1.44]	0.30
University vs. Ref.	0.89	[0.60, 1.31]	0.40	1.46	[1.00, 2.13]	0.05 *	1.76	[1.15, 2.70]	0.01 **
Bilingualism (Ref. = Monolinguals)									
Bilinguals vs. Ref.	0.86	[0.54, 1.38]	0.32	1.51	[0.97, 2.34]	0.06	1.32	[0.84, 2.06]	0.12
Referral Source (Ref. = Doctors)									
Parents vs. Ref.	1.73	[1.07, 2.80]	0.02 *	0.81	[0.51, 1.29]	0.18	1.44	[0.95, 2.20]	0.04 *
Teachers vs. Ref.	2.51	[1.32, 4.76]	0.004 **	0.72	[0.39, 1.33]	0.14	1.61	[1.09, 2.38]	0.02 *

Note. OR = odds ratio; CI = confidence interval. Reference groups: age = 0;7–3 years, gender = boys, SES = primary/secondary school, bilingualism = monolinguals, and referral source = doctors. SSD = speech sound disorder; DLD = developmental language disorder; LD = language disorder associated with biomedical conditions. * p < 0.05; ** *p* < 0.01.

## Data Availability

The data presented in this study are available on request from the corresponding author. The data are not publicly available due to privacy or ethical restrictions.
